# Plasticity of melanoma and EMT-TF reprogramming

**DOI:** 10.18632/oncotarget.1662

**Published:** 2013-12-19

**Authors:** Eugene Tulchinsky, J. Howard Pringle, Julie Caramel, Stéphane Ansieau

**Affiliations:** UMR INSERM 1052, CNRS 5286, Centre de Recherche en Cancérologie de Lyon, 28 rue Laennec, 69008 Lyon, France.; Department of Cancer Studies and Molecular Medicine, University of Leicester, RKCSB, LRI, Leicester, UK

For several decades, tumour progression has been considered as linear process involving clonal selection of most aggressive cell variants. Within the framework of this concept, multiple attempts have been made to identify genes specifically activated at late cancer stages and involved in cancer metastases. However, there are no experimental evidences supporting the existence of such purely “metastatic genes”, and metastatic behaviour is likely to be induced by the same mutationally-activated pathways that cause neoplastic transformation. In line with this view is the parallel model of tumour progression and metastases. It proposes that at least in some cancer types early metastatic spread is concomitant with oncogenic transformation, i.e. the same genetic insults are responsible for the tumourigenicity and metastatic dissemination [1, 2].

Epithelial-mesenchymal transition (EMT) is a reversible embryonic genetic program reactivated in cancer. EMT is controlled by several transcription factors, EMT-TFs (TWIST1, TWIST2, SNAIL1, SNAIL2, ZEB1, ZEB2, etc.). The initial view on EMT as just a process leading to enhanced cell motility and invasion was reconsidered in recent years. Aberrant activation of EMT programs in tumour cells affects cell proliferation, survival, drug resistance, tumourigenicity and stem cell-like features [3]. In particular, a proinvasive EMT-TF, TWIST1 cooperates with activated oncogenes by overriding oncogene-induced senescence [2]. These findings propose that EMT may act in concert with classical oncogenic pathways and contribute to different stages of tumourigenesis including tumor initiation, growth and spread.

We addressed the interrelationship between EMT-TF network and tumour-initiating pathways in malignant melanoma [4]. Melanoma represents an example of a cancer type solely dependent on the oncogenic pathways initiated by gain-of function mutations in BRAF, NRAS, GRM3 or MEK1/2 with all of them leading to the activation of MEK-ERK module. Melanocytic lineage is distinct from epithelium; it evolves from neural crest, an embryonic cell population regulated by EMT pathways. Our data show that normal melanocytes are positive for SNAIL2 and ZEB2, but negative for ZEB1 and TWIST1. In vitro activation of the MEK-ERK signalling results in the reversion of EMT-TF expression pattern, the downregulation of SNAIL2 and ZEB2 and upregulation of ZEB1/TWIST1. This reversible EMT-TF reprogramming is followed by the reduction in the expression of a master regulator of melanocytic lineage, microphthalmia-associated transcription factor (MITF), and repression of the downstream differentiation program. Moreover, switch to ZEB1 and TWIST1 is utterly required for the tumourigenic potential of BRAF in vitro and in vivo and for the increased invasiveness of BRAF-transformed melanocytes.

**Figure 1:**
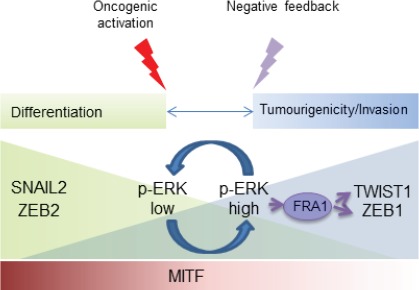
A scheme illustrating MEK-ERK-FRA1- regulated EMT-TFs switches during melanomagenesis These reversible switches impact on *MITF* expression and phenotypic plasticity in malignant melanoma.

Though the absolute majority of melanocytic lesions contain MEK-activating mutations, they exhibit a large degree of heterogeneity with regard to phospho-ERK immunopositivity. This is largely due to the activation of different negative feedback pathways in benign nevi and horizontal phase melanoma, and acquisition of bypass mechanisms in advanced cancer. Our analyses of melanoma samples have shown that EMT-TF reprogramming does exist in human melanoma. It strongly correlated with the phospho-ERK immunopositivity and poor patients' survival. Gradients of EMT-TF expression were observed in both the primary melanoma and independent or matched metastases. Namely, expression of ZEB2/SNAIL2 detected in superficial parts of primary tumours and cortical areas of lymph node metastases gradually decreased with the distance from the edge of the lesion. The expression of ZEB1 and TWIST1 was observed in deeper parts of primary tumours and medullary areas of nodal metastases and significantly correlated with phospho- ERK immunopositivity. The reestablishment of EMT-TFs gradients at secondary sites may indicate the existence of multiple waves of EMT-TF reprogramming, or reflect early metastatic dissemination. A so-called “rheostat model” proposes that MITF controls phenotypic switches between proliferative, differentiated and tumourigenic/invasive phenotypes in malignant melanoma [5]. Our data place the reversible switches in the EMT-TF network upstream of MITF in this model (Fig. 1). The reversibility of EMT-TF switches in melanoma is reminiscent of the reestablishment of epithelial morphology in carcinoma metastases [6]. In both cancer types, differentiated and tumourigenic states would be equally important for the accomplishment of the metastatic process.

An important player in the MEK/ERK/EMT-TF pathway is an AP1 transcription factor family member, FOS-related antigen 1 (FRA1). FRA1 is an effector of RAS pathway in different cancer types. In melanoma, it directly binds promoters of EMT-TF genes, and FRA1 depletion largely mimics an effect that MEK inhibition has on EMT-TF network. Reduced tumourigenic potential of melanoma cells with depleted FRA1 was rescued by ectopic expression of ZEB1 or TWIST1. Interestingly, a recent study has shown that FRA1 is activated by TWIST1 and SNAIL1 and serves as a gatekeeper of EMT/cancer stem cells program in breast carcinoma cells [7].

The existence of slowly proliferating, tumourigenic and drug-resistant cells within the tumour cell population represents an aggravating factor in failure of conventional anti-proliferative therapies. Sáez-Ayala M *et al*. proposed an alternative two-step treatment strategy based on methotrexate-mediated upregulation of MITF leading to the expression of melanocyte-specific tyrosinase gene in melanoma cells [8]. The second agent is the pro-drug, TMECG, which after being activated by tyrosinase, inhibits DHFR resulting in apoptosis. Given that FRA1 orchestrates EMT-TF reprogramming, an alternative method for switching MITF-dependent differentiation/transformation equilibrium in melanoma would be specific targeting of molecular pathways modulating FRA1 activity.
